# Doing What We Do, Better: Improving Our Work Through Systematic Program Reporting

**DOI:** 10.9745/GHSP-D-18-00136

**Published:** 2018-06-27

**Authors:** Irene Koek, Marianne Monclair, Erin Anastasi, Petra ten Hoope-Bender, Elizabeth Higgs, Rafael Obregon

**Affiliations:** a Bureau for Global Health, United States Agency for International Development, Washington, DC, USA.; b Department for Education and Global Health, Norwegian Agency for Development Cooperation, Oslo, Norway.; c United Nations Population Fund, New York, NY, USA.; d United Nations Population Fund, Geneva, Switzerland.; e National Institute of Allergy and Infectious Diseases, U.S. National Institutes of Health, Bethesda, MD, USA.; f United Nations Children's Fund, New York, NY, USA.

## Abstract

WHO has recently published program reporting standards to guide the type of information that reproductive, maternal, newborn, child, and related health programs should document to promote cross-program learning. We strongly encourage our partners and key stakeholders to make use of the new standards as part of their routine program reporting.

The world's 2030 Sustainable Development Goals (SDGs) put forth a broad, visionary, and ambitious agenda, including targets that require efficient, effective programs in communities and populations implemented at scale. Collectively, our organizations invest significantly in global health science and practice.

We all emphasize evidence-based learning, design, and implementation to improve the programs we support. There are several paths to improving the efficiency, effectiveness, and sustainability of programs; one path to improvement is cross-program learning. We often find, however, that program reports, when accessible, lack critical information necessary for reproducibility and adaptation to context. They also often lack information on key insights generated in the field that could be invaluable to other programs struggling with similar issues or seeking to implement similar interventions in a different context. Having accessible, systematic, comprehensive, and easily comparable data on program implementation—the how, why, who, and what—would facilitate learning, replication, validation, and scale up of interventions for different populations and environments.

Program reports often lack critical information necessary for reproducibility and adaptation to context.

For these reasons, we welcome the World Health Organization's (WHO's) recent publication on Program Reporting Standards (PRS) for sexual, reproductive, maternal, newborn, child, and adolescent health (SRMNCAH) programs.^1^ The objective of the PRS is “to provide guidance for complete and accurate reporting on the design, implementation, monitoring, and evaluation processes of SRMNCAH programs.”^1^ The PRS are intended for program managers and other staff who design, implement, and/or evaluate SRMNCAH programs, as well as implementation researchers who need to document important details of implementation and context. The PRS are comprised of 24 reporting items across 5 sections (Box). For example, the PRS indicate that implementation monitoring should include a description of the monitoring mechanisms; coverage/reach and drop-out rate; adaptations to programming; acceptability of programming; feasibility of programming; and factors affecting implementation.

BOX **WHO's Program Reporting Standards for Sexual, Reproductive, Maternal, Newborn, Child, and Adolescent Health: Reporting Items by Section**

1. Program overview
  - Rationale and objectives
  - Start and end date
  - Setting and context
  - Stakeholders
  - Funding source(s)
  - Theory of change and/or logic model
  - Human rights perspectives
2. Program components and implementation
  - Program planning
  - Piloting
  - Components/activities
  - Quality assurance mechanisms
3. Monitoring of implementation
  - Monitoring mechanisms
  - Coverage/reach and drop-out rate
  - Adaptations
  - Acceptability
  - Feasibility
  - Factors affecting implementation
4. Evaluation and results
  - Evaluation
  - Results
  - Costs
5. Synthesis
  - Lessons learned
  - Sustainability
  - Scalability
  - Possibilities for implementation in other settings

WHO's Program Reporting Standards provide guidance for complete and accurate reporting on the design, implementation, monitoring, and evaluation process of reproductive, maternal, newborn, child, and related health programs.

Gaps identified in program reporting between “what is learned in the field and what is communicated in scientific publications”^2^ and gray literature are long-standing. For example, the 2013 Population-Level Behavior Change Evidence Summit for Child Survival and Development, led by the United States Agency for International Development (USAID) and the United Nations Children's Fund (UNICEF), found systematic reporting gaps for social and behavior change programming and evidence for impact on child health.^3^ More than 100 experts reviewed hundreds of peer-reviewed articles and gray literature published since 1990 in order to develop recommendations for policy, programs, and research. They concluded that the basic information needed to assess the quality and context of much of the evidence they reviewed was lacking. Additionally, they concluded more broadly that the field of social and behavior change needs “to improve the way it reports successes and failures and collectively learns.”^3^ Systematic reviews and global guidance carried out by WHO^4^ in other program areas have reached similar conclusions. The United Nations Population Fund (UNFPA) also learned this through its efforts to review the availability and quality of emergency obstetric and newborn care and to determine the drivers for successful implementation of policies and programs.^5^

WHO's extensive and collaborative process to develop these PRS originated in a shared understanding of the need for more “action-oriented” information in the peer-reviewed and gray literature. Kågesten et al.^2^ identified and reviewed several other existing reporting guidelines in their work developing the PRS. While the existing reporting guidelines are considered relevant and have some overlap in reporting items with the new WHO PRS, they were mostly developed for translation or synthesis of research findings and fail to include key aspects of context; details of intervention or program design, the development process, implementation, or delivery; and evaluation processes and outcomes of particular relevance to policymakers, program implementers, and their partners.^2^ The new PRS provide guidance on achieving more complete and accurate documentation of those processes and outcomes, including important factors such as context, sustainability, scalability, and stakeholder involvement.

The new PRS place emphasis on context, which is needed to understand how programs are delivered, key factors accounting for observed performance, and issues that may be important in its potential replication and/or scale up, including factors that can vary over the program's planned timeframe. Currently, contextual factors such as norms, behaviors, social networks, existing policies, and health system capacity are not included in routine reporting, though implementation success often depends on such factors. With organizations supporting and implementing programs across a wide range of different contexts, this information is critical to better understand both successful and failed programming; replicate and/or adapt programs as needed; and scale up effective interventions. Reporting items on sustainability and scalability can help ensure that these aspects are considered, as appropriate, from the beginning of implementation and are continually reflected upon throughout implementation. The process of stakeholder involvement and local ownership of programs needed for sustainability is considered vital by donors and United Nations (UN) agencies supporting governments; however, very little is reported on the success or failure of these processes. Use of PRS would allow for a consistent documentation of these experiences.

From an implementation perspective, these standards are practical and easily applicable, though some adaptation may be required for larger programs.^3^ Widespread use of these standards for reports and publications should much improve the usefulness of program reporting, adding essential information on context and facilitating more relevant cross-country learning. The new PRS have the advantage of bringing all of the reporting items together in a single document and providing guidance on definitions and quality standards of the reporting items. In other words, what WHO is proposing is facilitating a more coherent and more systematic way of reporting, with the specific goal of better serving program learning needs.

WHO's PRS ensure a comprehensive approach to documentation of the program development cycle, from design to evaluation and results. The ability to find all the needed information in a single document—and to be able to compare or collate this information across programs—represents a relatively modest change in existing processes that could, nevertheless, result in significant benefits, not only for program managers and implementers but also for program participants/beneficiaries and the larger global health research, policy, and practice communities.

One key consideration that Kågesten et al. highlight in their article is the importance of uptake and use of the PRS. Given that the PRS are intended for use by any implementing partner, dissemination will be important through a range of channels and through different partners such as donors, UN, international NGOs, and the private sector, among others. Agencies and organizations could help in its uptake by incorporating PRS as a strong recommendation or as a standard element of contractual agreements and programming guidance provided to partners, country offices, and other stakeholders. A second but no less important piece will be ensuring the availability of the reports so the information can be widely accessed and shared. Many agencies and organizations already do this.

Agencies and organizations can help with uptake of the Program Reporting Standards by incorporating them into contractual agreements and programming guidance provided to partners and country offices.

*Global Health: Science and Practice* is an important venue for sharing programmatic experiences with sufficient space for discussion of context, implementation, and experiences. A key consideration moving forward is how to make sure that this tool for improving the way we report on and communicate about programs—for providing more thorough and accurate information on program successes and failures—is widely available, shared in other publications, and, ultimately, used. Progress in global public health depends on the ability of the field to share, learn from, and build on each other's programs, including understanding what did or did not work as well as why and in what contexts. Accurate, complete, and comparable reporting will help make this possible.

We are pleased that WHO is taking concrete steps to address these gaps and to harmonize and strengthen reporting across the broad field of SRMNCAH programming. The new standards apply beyond the field of SRMNCAH and are an important contribution to document progress and achievements toward the SDGs. We strongly recommend our partners and key stakeholders in the field of global health and development take ownership and systematically make use of the new reporting standards as part of their routine program reporting.

## Notes

**Peer Reviewed**

**Funding:** None.
